# Emotional Distress of Patients at End-of-Life and Their Caregivers: Interrelation and Predictors

**DOI:** 10.3389/fpsyg.2018.02199

**Published:** 2018-11-06

**Authors:** Ana Soto-Rubio, Marian Perez-Marin, Jose Tomas Miguel, Pilar Barreto Martin

**Affiliations:** ^1^Department of Personality, Assessment and Psychological Treatments, Faculty of Psychology, University of Valencia, Valencia, Spain; ^2^Department of Methodology of the Social Sciences, Faculty of Psychology, University of Valencia, Valencia, Spain

**Keywords:** burden, end-of-life, family caregiver, emotional well-being, cognitive impairment

## Abstract

**Background:** Patients at the end of life and their families experience a strong emotional impact. The well-being of these patients and that of their family caregiver are related.

**Aim:** To study the variables related with the emotional well-being of patients with and without cognitive impairment at the end of life and that of their primary family caregivers.

**Design:** Cross- sectional study.

**Participants**: Data was collected from 202 patients at the end of life with different diagnosis (COPD, cancer, and frail elderly) as well as from their respective 202 primary family caregivers.

**Results:** Structural equation models indicated that the emotional state of the patients was best predicted by their functional independence and the burden of their family caregivers. In addition, the emotional state of the primary family caregiver was predicted by their burden and not by the cognitive state or the functional independence of the patient. Nevertheless, the burden of the family caregiver, which is the only variable predicting both the emotional state of the patient and that of the caregiver, was directly related with the functional independence of the patient and indirectly with the patient’s cognitive state.

**Conclusion:** The family caregiver’s burden is an important factor to take into consideration when aiming to reduce the emotional distress of patients at the end of life with different diagnosis -whether or not they present significant cognitive impairment- and that of their family caregivers.

## Introduction

The World Health Organization defines palliative care as “an approach that improves the quality of life of patients and their families facing the problem associated with life-threatening illness, through the prevention and relief of suffering by means of early identification and impeccable assessment and treatment of pain and other problems, physical, psychosocial, and spiritual” (Who, 2005).

The physical aspects of discomfort and suffering have been extensively studied in the context of palliative care (Cherny et al., 2015). In this context, it has also been highlighted the importance of the psychological and social aspects of suffering, when aiming to reduce the distress of these patients and that of their families (Cassell, 1998; Callahan, 2000; Who, 2005; Bennett and Shepherd, 2013; Grassi et al., 2015; Cipolletta et al., 2016, 2017; Fu et al., 2017).

The accurate assessment of needs is of capital importance in order to fulfill one of the main objectives of palliative care: the reduction of suffering of patients and their families (Sepúlveda et al., 2002). These needs have been considerably studied in the last 20 years, and based on these studies specific assessment instruments have been developed (Davis et al., 2016; Soto-Rubio A.L. et al., 2017). Nevertheless, the assessment of needs can be particularly challenging when the patient is no longer able to communicate (Sampson et al., 2006, 2015). Often, in end-of-life situations, the patient is unable to communicate because of fatigue, weakness, and a reduced level of awareness due to drugs to alleviate pain (Herr et al., 2006). At the same time, the advanced stage of some pathologies is associated to cognitive impairment (Liszewski et al., 2004; Cysique et al., 2006; Green, 2006; Pandharipande et al., 2013; Cosgrove and Alty, 2018). The severe physical and cognitive impairment of many patients hampers the assessment and treatment of their psychological needs (Sampson et al., 2006; Davis et al., 2016), especially those related to their emotional well-being.

Despite the obstacles to communication that may arise in end-of-life situations, the emotional well-being of the patients must be safeguarded (Oczkowski et al., 2016). In cases where the patient presents difficulties communicating verbally, there are behavioral parameters that can be observed and registered in order to assess the patient’s level of comfort/discomfort, such as facial expressions and posture and body language (Soto-Rubio A.L. et al., 2017). The European Association for Palliative Care recommends for these cases the use of observational scales (Sampson et al., 2015).

The strong emotional impact experienced by patients at the end of life and their families has been widely described in previous research (Baum et al., 2001; Pessin et al., 2002; Barreto and Martínez, 2003; Krikorian et al., 2012). Several studies address the needs of these families: social, physical, spiritual, emotional, and financial (Given et al., 2012; Chochinov et al., 2016; Fombuena et al., 2016). The emotional distress of the family caregivers in this context is characterized by symptoms of anxiety and depression (Toseland et al., 1995; Raveis et al., 1998, 2000; Sherwood et al., 2005; Northouse et al., 2012).

Also, the caring tasks and the restrictions that come with them usually cause discomfort in the caregiver (Buhse, 2008), which can lead to caregiver burden: a multidimensional response to psychological, physical, social, and financial stressors related to the caregiving experience (Zarit et al., 1980).

Previous research in the context of palliative care has provided evidence supporting a relationship between the patient’s emotional state and that of their family caregiver (Hodges et al., 2005; Bishop et al., 2007; Segrin et al., 2007; Hagedoorn et al., 2008). For this reason, it is of great importance to study the specific way in which the emotional state of these patients and that of their family caregivers are related, taking into consideration the possible influence that the cognitive state of the patient may be having. Moreover, this relationship should be taken into consideration in the design and implementation of interventions that aim to reduce the suffering of patients and families in the end-of-life context.

The present study aims to study the variables related with the emotional well-being of patients with and without cognitive impairment at the end of life and that of their primary family caregivers. More specifically, it analyses the relationships among the cognitive state, functional independence and emotional state of the patient, and the burden and emotional state of the primary family caregiver.

## Materials and Methods

### Sample

All data was collected between September 2015 and September 2016. Cross- sectional data was collected from 202 patients at the end of life as well as from their respective 202 primary family caregivers. The age of the patients ranged from 43 to 99 years (*M* = 76.46 years; *SD* = 9.850), and 68.8% were men. The age of the family caregivers ranged from 22 to 92 years (*M* = 61.46 years; *SD* = 13.521), and 22.8% were men.

All patients were being attended in a Palliative Care Unit at the moment of assessment. The patients’ inclusion criteria were:

- Being judged by the medical team to be in an end-of-life situation, in accordance with the criteria established by the Spanish Society of Palliative Care (Sociedad Española de Cuidados Paliativos [SECPAL], 2018).
- The main diagnosis included at least one of the following: chronic obstructive pulmonary disease (COPD), cancer or frail elderly.

The family caregivers’ inclusion criterion was:

- To be the primary family caregiver of a patient participating in the study. In the present study, family caregiver is defined as “the member of the family that assume the main tasks of caregiving, and take care of the patient most of time, or during a longer time than other members of the family.”

The exclusion criterion for the family caregivers was to present cognitive decline.

Data was collected through an interview that took place the first week of admission in the palliative care unit of one of the hospitals participating in the study. All participants from the study signed an informed consent, in accordance with the Declaration of Helsinki. In the cases where the patients presented cognitive impairment, it was their legal tutor who signed an informed consent. The assessment protocol was approved by the Ethics Committee of the University of Valencia (H1385291905651).

### Measures

Data about socio-demographic features from patients and family caregivers (e.g., age, gender, marital status, education, kinship between patient, and family caregiver) was registered. Also, information on the following aspects was collected.

#### Functional Independence of the Patient

This variable was assessed using the Barthel’s scale (Mahoney and Barthel, 1965), adapted to Spanish population (Baztán et al., 1993). The scores for this scale range from 0 to 100 (0 to 90 in case of wheelchair bound people), providing five levels of dependency (Mahoney and Barthel, 1965).

#### Cognitive State of the Patient

To assess this variable, it was used the Short Portable Mental Status Questionnaire (SPMSQ) (Pfeiffer, 1975) validated for Spanish population (Martínez et al., 2001). It is a 10 items questionnaire. Its total score range from 0 to 10. In the present study, a score ≤ 5 is rated as “non-significant cognitive impairment,” and a score > 5 is rated as “significant cognitive impairment” (the cut-off point is 6 instead of 5 for people with no elementary studies).

#### Emotional State of the Patient (ESP)

In order to assess the emotional state of the patient, the following tools were used: Hospital Anxiety and Depression Scale (HADS) (Zigmond and Snaith, 1983) adapted to Spanish population (Tejero et al., 1986): this questionnaire was used in patients without significant cognitive impairment to assess their emotional state (ESP). It includes 14 items that assess the main emotional and cognitive indicators of anxiety and depression. The total score ranges from 0 to 42. It provides two categories of global emotional state: <21 = absence of clinically significant emotional distress, >20 = presence of clinically significant emotional distress. In this study we will refer to this variable as ESP-HADS.

Discomfort Observation Scale (DOS) (Soto-Rubio A.L. et al., 2017): this scale was used to assess the emotional state (ESP) in patients with significant cognitive impairment. It presents nine items, each one describing a behavioral indicator whether of distress or of well-being. The scale is filled by a member of the medical staff of the palliative care unit, marking the presence or absence of each behavioral indicator. It offers scores from 0 to 9, higher scores meaning higher levels of discomfort. A score lower than 5 rates as a low level of discomfort, whereas a score equal or higher than 5 rates as a high level of discomfort. In this study we will refer to this variable as ESP-DOS.

#### Emotional State of the Primary Family Caregiver (ESF)

To assess this variable, it was used the HADS already described in this article. We will refer to this variable as ESF-HADS.

#### Burden of the Primary Family Caregiver

The Caregiver’s Burden Scale (Zarit et al., 1980): this scale contains 22 items rated on a five-point Likert scale, from 0 (“never”) to 4 (“nearly always”). It offers scores from 22 to 110, higher scores reflecting greater burden, and it is suitable for different populations (Hébert et al., 2000). It offers three levels of burden taking into account the total score: 22–46 = no burden, 47–55 = light burden, 56–110 = intense burden.

### Statistical Analyses

Zero-order correlations have been calculated in SPSS 24. Correlation comparisons were also calculated between the two samples. Several structural equation models have been estimated and tested in Mplus 7.0 (Muthén and Muthén, 1998–2011). The structural model has been tested simultaneously in a multigroup routine in patients with and without significant cognitive impairment. The measurement invariance models are nested and their relative plausibility (fit) must be assessed. The plausibility of the structural models was assessed using: (a) the chi-square statistic (Kline, 2015); (b) the comparative fit index (CFI) of more than 0.90 (Bentler, 1990) (and, ideally, greater than 0.95) (Hu and Bentler, 1999); (c) Akaike Information Criterion (AIC) to compare models, with lower values indicating better fit; and (d) the root-mean-squared error of approximation (RMSEA) of 0.08 or less (and, ideally less, than 0.05) (Hu and Bentler, 1999). Nested models, as the ones in the invariance routine, can be compared with two rationales (Little, 1997): the statistical and the modeling one. The statistical rationale compares the chi-squares of the alternative models, with non-significant values suggesting multi-group equivalence or invariance. However, this statistical approach has been usually combined with the comparison of fit indices (Cheung and Rensvold, 2002). This practical or modeling approach, advocated among others by Little (1997), states that if a parsimonious model (such as the ones that posit invariance) evinces adequate levels of practical fit, then the sets of equivalences are considered a reasonable approximation to the data. Practical fit is usually determined with CFI differences. CFI differences lower than 0.01 (Cheung and Rensvold, 2002) or 0.05 (Little, 1997) are usually employed as cut-off criteria.

## Results

Descriptive statistics for variables of cognitive state, functional independence, and emotional state of the patient, as well as burden and emotional state of the primary family caregiver are presented in Table 1.

**Table 1:** Descriptive statistics for main variables of study.

	Frequency	Percentage	Valid percentage
Emotional state of the patient (HADS)			
Clinically significant emotional distress	31	15.3	23.7
Non-clinically significant emotional	100	49.5	76.3
distress	131	64.9	100
Total			
Emotional state of the patient (DOS)			
High	33	16.3	45.8
Low	39	19.3	54.2
Total	71	35.6	100
Cognitive state of the patient			
Non-significant cognitive impairment	131	64.9	64.9
Significant cognitive impairment	71	35.1	35.1
Total	202	100	100
Functional independence of the patient			
Total dependency	80	39.6	39.8
Severe dependency	16	7.9	8
Moderate dependency	14	6.9	7
Mild dependency	76	37.6	37.8
Independency	15	7.4	7.5
Total	201	99.5	100
Emotional state of the Caregiver (HADS)			
Clinically significant emotional distress	47	23.3	23.6
Non-clinically significant emotional	152	75.2	76.4
distress	199	98.5	100
Total			
Burden of the caregiver			
No burden	120	59.4	60
Light burden	35	17.3	17.5
Intense burden	45	22.3	22.5
Total	200	99	100

### Correlations

We undertook preliminary correlational analyses to test if the correlation between the burden of the primary family caregiver and the emotional state of the patient was of the same direction and magnitude when estimated in patients with and without significant cognitive impairment. That is, the zero-order correlation between the emotional state of the patient and the burden of the family caregiver was estimated for patients without cognitive impairment (*r* = 0.23, *p* = 0.007), and for patients with cognitive impairment (*r* = 0.29, *p* = 0.005), and both correlations were statistically significant and positive. Then, both correlations were compared to test the null hypothesis of equal correlation, and this analysis was not statistically significant (*z* = -0.41, *p* > 0.05). Therefore, there is no evidence that the positive relationship between the emotional state of patient and the burden of the caregiver is different due to the change in the instrument used to measure patients’ emotional state.

### Structural Models

**FIGURE 1:**
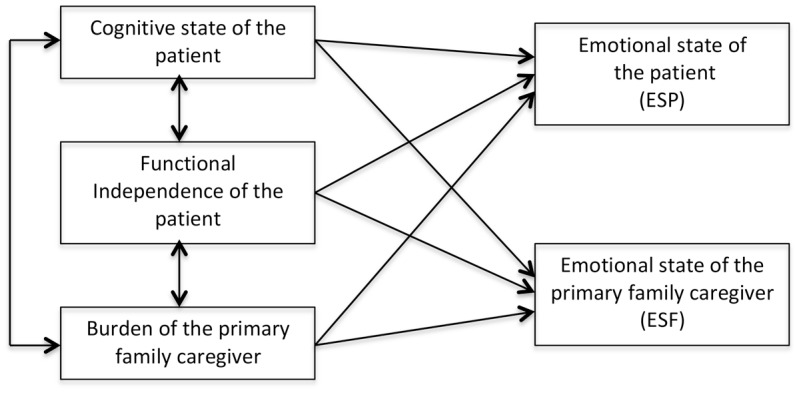
Theoretical structural model.

An *a priori* structural model was specified to relate the variables of interest (Figure 1). This model was tested in the sample of patients without cognitive impairment, the largest one, and fitted the data reasonably well [χ^2^(1) = 2.59, *p* = 0.10. CFI = 0.979, RMSEA = 0.112, 90% CI(0.000, -0.289), and AIC = 0.594]. However, this model is not parsimonious enough as the RMSEA shows and some relations were not statistically significant.

**FIGURE 2:**
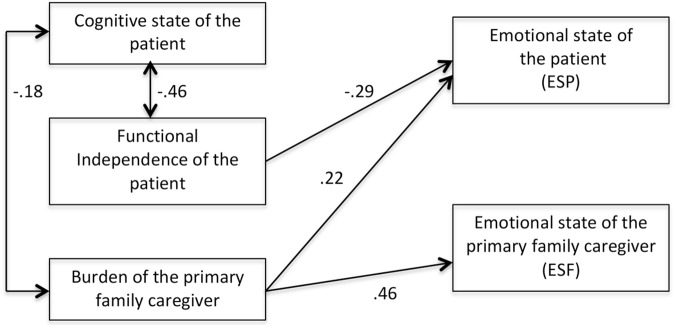
Final model estimated in the sample of patients without cognitive impairment.

We removed the statistically non-significant relations and tested this new, more parsimonious, model. This model fitted the data very well: χ^2^(5) = 6.39, *p* = 0.26. CFI = 0.981, RMSEA = 0.047, 90% CI(0.000, -0.138), and AIC = -3.60. Moreover, a statistical comparison of both models showed no statistically significant differences [χ^2^(4) = 3.81, *p* = 0.43], the AIC for the more parsimonious model was lower than the AIC for the initial model and the CFI improved in the re-specified model. Accordingly, we decided to retain this model (Figure 2) as the best fitting model. This new, modified model, has then been analyzed with a multigroup routine. That is, the best-fitting model has been simultaneously tested in the samples of patients with and without cognitive impairment. This multigroup routine tries to test if the same relationships (and their magnitude) holds for both samples even tough the instrument of measurement for emotional state of the patients with and without cognitive impairment is not the same. The multigroup routine starts with a multisample model simultaneously tested in both samples with no constraints across groups being made. This unconstrained multisample model had a very good fit: χ^2^(10) = 13.63, *p* = 0.23. CFI = 0.969, RMSEA = 0.063, 90% CI(0.000, -0.158), and AIC = -6.36. Given that the model fitted the data well for both samples, a more parsimonious model, with the three relationships among the predictors and the criteria constrained to be equal was estimated. This constrained model tests for the hypothesis of same magnitude in the relationships among the constructs of interest in the samples of patients with and without cognitive impairment. If model fit does not deteriorate, and ideally it is not statistically different from the fit in the unconstrained model, then this is evidence of same relationships holding for both samples. This constrained model fitted the data well: χ^2^(13) = 14.69, *p* = 0.32. CFI = 0.986, RMSEA = 0.038, 90% CI(0.000, -0.158), and AIC = -11.30. Indeed, model fit did improve, as CFI is larger and RMSEA and AIC lower than in the unconstrained model. A robust chi-square difference test was not statistically significant [χ^2^(3) = 0.784, *p* = 0.85] reinforcing the evidence of equal relations in both samples. Therefore, the estimates in Figure 2 adequately represent both samples.

## Discussion

The main contribution of this study is the empirical proposal of a model that reflects the main predictors of the patient’s emotional well-being at the end of life and that of their main family caregiver. We observed in the proposed structural equation model how the overburden of the family caregiver act as a predictor of the emotional state of both patient and family caregiver. In this model, the relevance of the level of burden of the family caregiver is pivotal, since it directly predicts the emotional discomfort of the patient and the caregiver, even more than the level of functional independence of the patient or their level of cognitive impairment. These results suggest that reducing the family caregiver’s level of burden may contribute to the reduction of their own emotional distress and that of the patients they are taking care of. In this sense, our study provides useful data on the emotional well-being of the family system, specifically on the patient-caregiver dyad, highlighting the importance of the mutual influence between the well-being of the patient and that of the caregiver, as a starting point for the application of the model in future studies (Li and Loke, 2014).

An advantage of the model proposed in this study is that it predicts the emotional distress of the patient at the end of life and their main family caregiver whether the patient presents significant cognitive impairment or not. Therefore, we think our research provides new and useful knowledge in this sense.

Although the patient’s functional independence should be considered when promoting their own well-being and that of their families, our study suggest that the family caregiver’ burden might be equally important for this purpose. This is particularly interesting because there are effective interventions that might be implemented to reduce the overburden of the caregiver (Sörensen et al., 2002; Etters et al., 2008), whereas an intervention to reduce the patient’s functional dependence is not always feasible.

Data supporting the finding of the overburden of the family caregiver predicting the caregiver’s own emotional well-being and that of the patients has been found in previous research with patients at the end of life with a single specific diagnosis, like frail elderly (Soto-Rubio A. et al., 2017). The present study includes patients with different diagnosis (cancer, COPD, and frail elderly) while it takes into consideration their cognitive states, which makes our findings applicable to more diverse end-of-life settings.

One possible limitation of this study is that the patient’s emotional distress has been measured with two different instruments, depending on the cognitive status of the patients. However, both the correlation comparison analyzes and the comparison of the different structural equation models provide data in favor of the use of these two instruments in this context, since the observed relationships between the variables are the same.

## Conclusion

This study suggests the need of implementing intervention programs in order to reduce the emotional distress of the patients at the end of life and their family caregivers, as well as to prevent the family caregivers’ overburden. Furthermore, the family caregiver’s overburden stands out as an important factor when aiming to reduce the emotional distress of patients at the end of life and their families, and this applies whereas the patient presents significant cognitive impairment or not. Our findings preliminarily suggest that reducing the family caregiver’s burden may also contribute to the reduction of the emotional distress of both patients and caregivers. In consequence, those interventions aimed to reduce the emotional distress of patients at the end of life and of their family caregivers should pay especial attention to indicators of overburden of the family caregiver, facilitating at the same time its prevention and reduction.

In this sense, future research regarding the reduction of emotional distress in the end-of-life context should take into consideration the prevention and reduction of overburden in family caregivers.

## Data Availability

The raw data supporting the conclusions of this manuscript will be made available by the authors, without undue reservation, to any qualified researcher.

## Author Contributions

AS-R contributed to the writing of the article and collection of data. MP-M contributed to the writing of the article and supervision of the research. JT contributed to the writing of the article and methodological analysis. PB contributed to the writing of the article and supervision of the research.

## Conflict of Interest Statement

The authors declare that the research was conducted in the absence of any commercial or financial relationships that could be construed as a potential conflict of interest.
